# MRSA PCR improves sensitivity of detection of colonization in neonates

**DOI:** 10.1017/ash.2023.302

**Published:** 2023-09-29

**Authors:** Nahid Hiermandi, Catherine Foster, Krystal Purnell, James Dunn, Judith Campbell, Lucila Marquez

## Abstract

**Background:** Neonates colonized with methicillin-resistant *Staphylococcus aureus* (MRSA) are at high risk of developing life-threatening MRSA infection. Due to lack of evidence, national guidelines do not currently recommend a specific methodology for detecting MRSA colonization. We hypothesize that surveillance for MRSA colonization via polymerase chain reaction (PCR) is superior to culture for the detection of colonization. **Methods:** In this retrospective study, we compared results of MRSA surveillance by 2 methodologies, culture and PCR, after implementation of an MRSA surveillance and decolonization protocol in the Texas Children’s Hospital Pavilion for Women, a 42-bed neonatal intensive care unit. MRSA colonization of 3 body sites via the 2 methodologies was assessed from June 2017 through December 2020. All neonates were screened for MRSA upon admission to the NICU and weekly thereafter until MRSA-positive or discharged. Swab specimens were initially tested by PCR (Xpert MRSA NxG, Cepheid) and when MRSA-positive reflexed to culture to recover the organism for further characterization. This study was approved through the Baylor College of Medicine Institutional Review Board. **Results:** During the study period, 2,351 neonates were assessed for MRSA colonization by PCR; 81 (3.4%) infants were PCR positive (Fig. 1). Of those 81, 57 (70.4%) had concordant MRSA PCR and culture results, and 24 (29.6%) were MRSA PCR positive but no isolate was recovered in culture. Also, 8 specimens were indeterminate by PCR. However, 1 infant who was negative by culture but was PCR positive developed an MRSA orbital infection. Compared to PCR, the overall sensitivity of MRSA culture was 70.4% (range, 57.7%–80.8%, depending on the year) (Table 1). **Conclusions:** PCR is more sensitive than culture for detecting MRSA colonization in neonates. Utilizing a PCR method enhances the ability to identify MRSA colonized infants more readily and allows for prompt initiation of infection control interventions including isolation precautions and decolonization strategies. Reflex to culture remains important for strain characterization during outbreak investigations and for additional susceptibility testing. Resource utilization and cost–benefit analyses should be done in future studies to influence changes in national guidelines for the control of *Staphylococcus aureus* colonization and infection in neonatal intensive care units.

**Figure:**
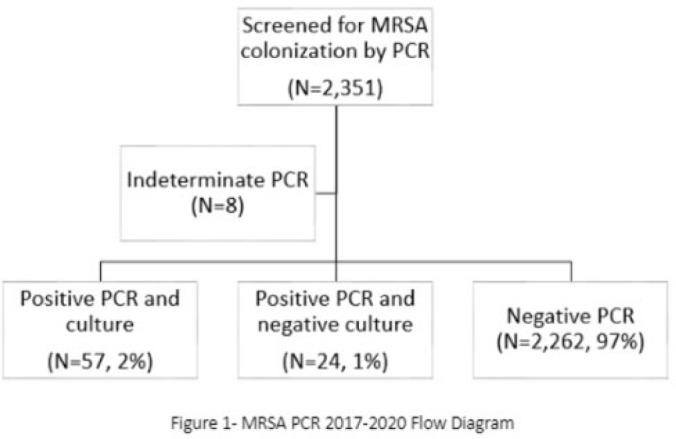

**Figure:**
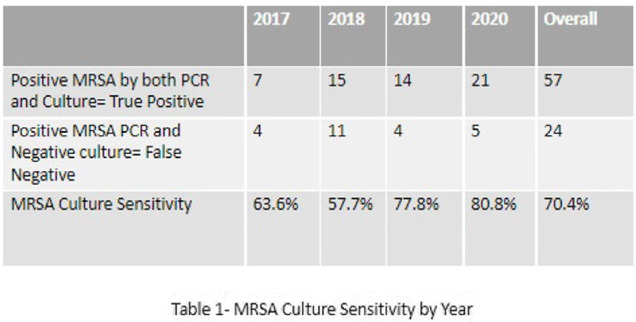

**Disclosures:** None

